# The effect of donor against recipient one-way HLA mismatch on liver transplantation outcomes from a multicenter registry analysis

**DOI:** 10.1038/s41598-023-49178-y

**Published:** 2023-12-15

**Authors:** Sunghae Park, Young Rok Choi, Dong Jin Joo, Young Kyoung You, Bong-Wan Kim, Yang Won Nah, Jai Young Cho, Tae-Seok Kim, Geun Hong, Man Ki Ju, Suk-Won Suh, Jae Do Yang, Pyoung Jae Park, Jaehong Jeong, Ju Ik Moon, Dong-Sik Kim, Jinsoo Rhu, The Korean Organ Transplantation Registry Study Group

**Affiliations:** 1 Department of Surgery, Samsung Medical Center, Sungkyunkwan University, School of Medicine, 50 Irwon-Dong, Gangnam-gu, Seoul, 135-710 Korea; 2 Department of Surgery, Seoul National University College of Medicine, Seoul National University Hospital, Seoul, Korea; 3 Department of Surgery, Yonsei University College of Medicine, Seoul, Korea; 4 Department of Surgery, College of Medicine, The Catholic University of Korea, Seoul, Korea; 5 Department of Liver Transplantation and Hepatobiliary Surgery, Ajou University School of Medicine, Suwon, Korea; 6 Department of Surgery, Ulsan University Hospital, University of Ulsan College of Medicine, Ulsan, Korea; 7 Department of Surgery, Seoul National University Bundang Hospital, Seoul National University College of Medicine, Seoul, Korea; 8 Department of Surgery, Dongsan Medical Center, Keimyung University School of Medicine, Daegu, Korea; 9 Department of Surgery, Ewha Womans University Medical College, Seoul, Korea; 10 Department of Surgery, Yonsei University Gangnam Severance Hospital, Seoul, Korea; 11 Department of Surgery, College of Medicine, Chung-Ang University, Seoul, Korea; 12 Department of Surgery, Jeonbuk National University Hospital, Jeonju, Korea; 13 Department of Surgery, Korea University Guro Hospital, Korea University College of Medicine, Seoul, Korea; 14 Department of Surgery, School of Medicine, Soonchunhyang University, Bucheon, Korea; 15 Department of Surgery, Konyang University Hospital, Daejeon, Korea; 16 Department of Surgery, Korea University College of Medicine, Seoul, Korea

**Keywords:** Liver, Risk factors

## Abstract

Donor against recipient one-way Human leukocyte antigen (HLA) mismatch (D → R one-way HLA MM) seemed strongly associated with graft-versus-host disease (GVHD). The aim of this study is to investigate the relevance of D → R one-way HLA MM in outcome of liver transplantation (LT). We retrospectively analyzed 2670 patients in Korean Organ Transplantation Registry database between April 2014 and December 2020. The patients were categorized into two groups whether D → R one-way HLA MM or not and evaluated the outcomes of LT between the two groups. 18 patients were found to be D → R one-way HLA MM. The incidence of GVHD (0.3% vs. 22.2%, *p* < 0.001) and mortality rate (11.6% vs. 38.9%, *p* = 0.003) was much higher in D → R one-way HLA MM group. D → R one-way HLA MM at 3 loci was seemed to be strongly associated with the incidence of GVHD (OR 163.3, *p* < 0.001), and found to be the strongest risk factor for patient death (HR 12.75, *p* < 0.001). Patients with D → R one-way HLA MM at 3 loci showed significantly lower overall survival (*p* < 0.001) but there were no significant differences in rejection-free survival and death-censored graft survival. D → R one-way HLA MM at 3 loci not only affects the overall survival of LT patients but also the incidence of GVHD.

## Introduction

Human leukocyte antigen (HLA) compatibility between donor and recipient is closely related to the clinical outcomes of most solid organ transplantation. However, unlike other solid organ transplantation, the influence of HLA compatibility in liver transplantation (LT) remains unclear^1^. It is obvious that higher HLA compatibility may decrease the probability of acute or chronic rejection after LT but some previous studies demonstrated “dualistic effect” of HLA compatibility on LT which can lead to adverse effect^2,3^. Therefore, nowadays, matching HLA type of donor and recipient is not routinely required before LT.

Graft-versus-host disease (GVHD) rarely occurs after LT, with an incidence rate of 0.1–2.0%^4,5^. However, it can result in devastating outcome, since the mortality rate of GVHD after LT has been reported to be up to 85%^6,7^. It is known that GVHD is caused by immunocompetent donor lymphocytes, which recognize the recipient’s antigen and amplify the immune response after transplantation^7^. Because of its low incidence rate and nonspecific symptoms, effective diagnostic tools or treatment options for GVHD have not yet been clearly established. To date, known risk factors for GVHD include recipient and donor age, glucose intolerance, LT for hepatocellular carcinoma and HLA compatibility^8^. Several studies suggested that donor against recipient one-way HLA mismatch (D → R one-way HLA MM) may highly increase the incidence of GVHD after living donor LT but could not give enough evidence due to the small case number of GVHD.

In this study, we investigate the relevance of D → R one-way HLA MM to GVHD as well as graft and patient survival in LT by analyzing Korean Organ Transplantation Registry (KOTRY) database.

## Results

### Patient characteristics

**Figure 1:**
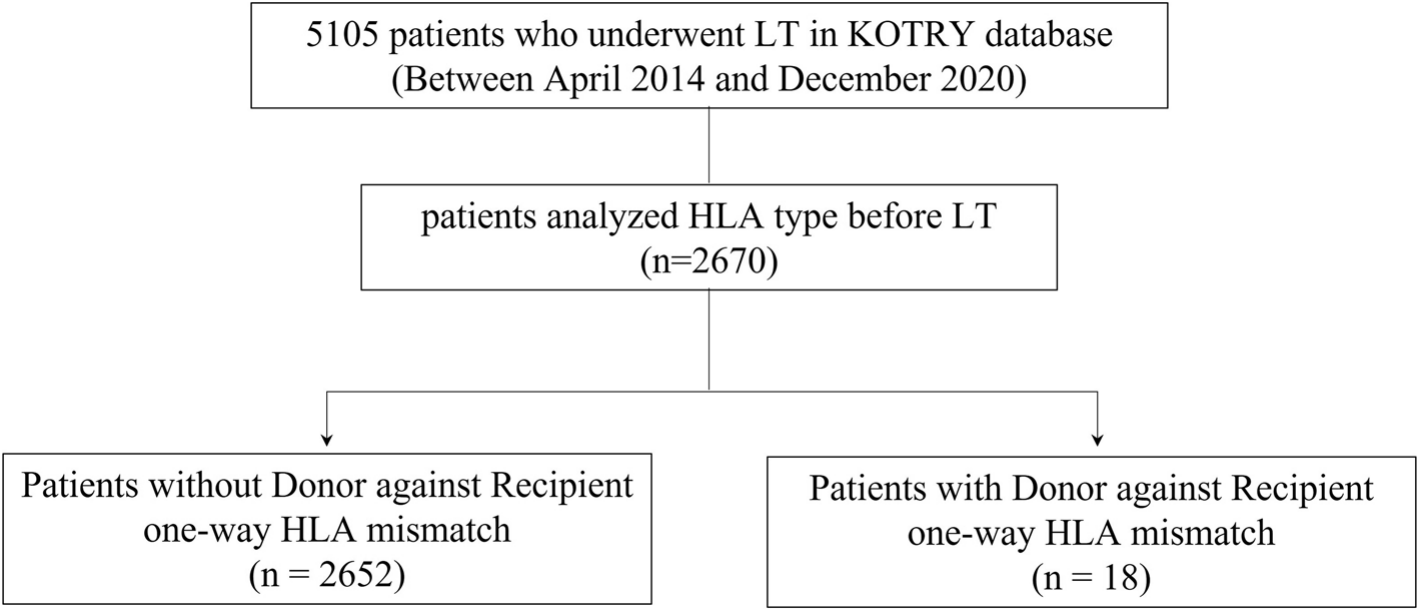
Flow diagram of patient selection criteria.

Patient selection criteria is shown in Fig. 1. Among 5105 patients who underwent LT between April 2014 and December 2020 in KOTRY database, 2670 patients with HLA typing data were included in this study. A total of 18 patients with D → R one way HLA MM were compared with 2652 patients without D → R one-way HLA MM as control group (no D → R one way HLA MM group).

Table 1 summarizes the patient characteristics between the D → R one way HLA MM group and no D → R one way HLA MM group. All patients in D → R one way HLA MM group underwent living donor LT, whereas in the control group, 75.1% of patients underwent living donor LT (*p* = 0.011). In D → R one way HLA MM group, a higher proportion of donors were offspring of recipients compared to the control group (47.7% vs. 83.3%, *p* = 0.002). In addition, there was significant difference in transplanted graft type between the two groups (*p* = 0.045). 83.3% of recipients in D → R one way HLA MM group received right hemiliver graft while no patient received whole liver graft from their donors. Median warm ischemic time during LT was longer in D → R one way HLA MM group (35.3 min vs. 50.0 min,* p* = 0.025). Among 2670 LT recipients, total 11 patients underwent GVHD after LT. The incidence of GVHD (0.3% vs 22.2%, *p* < 0.001) and death (11.6% vs. 38.9%, *p* = 0.003) was much higher in D → R one way HLA MM group compared to the control group.

**Table 1:** Patient characteristics.

	no D → R one way MM (n = 2652)	D → R one way MM (n = 18)	*p* value
Recipient characteristics			
Age (years, median)	55.5 (48.8–61.3)	54.4 (45.8–64.7)	0.742
Sex (n, % male)	1846 (69.6)	11 (61.1)	0.6
Weight (kg, median)	65.1 (56.5–73.8)	64.1 (56.0–71.3)	0.666
BMI (kg/m^2^, median)	23.6 (21.3–26.3)	23.3 (21.1–27.3)	0.828
Etiology (n, %)			0.438
HBV	1312 (49.5)	6 (33.3)	
HCV	156 (5.9)	1 (5.6)	
Alcoholic	682 (25.7)	6 (33.3)	
Cryptogenic	163 (6.1)	2 (11.1)	
Autoimmune	71 (2.7)	0 (0.0)	
PBC/PSC/SSC	54 (2.0)	0 (0.0)	
Congenital bile duct disorder	81 (3.1)	1 (5.6)	
Other	133 (5.0)	2 (11.1)	
Liver cancer (n, %)			0.725
HCC	1302 (49.1)	10 (55.6)	
CCC	8 (0.3)	0 (0.0)	
HCC & CCC	25 (0.9)	0 (0.0)	
Other malignancy	4 (0.2)	0 (0.0)	
Reason for primary LT			1.000
Acute liver failure	114 (4.3)	0 (0.0)	
Acute on chronic liver failure	123 (4.6)	0 (0.0)	
Liver cirrhosis	1076 (40.6)	8 (44.4)	
Liver cancer	1339 (50.5)	10 (55.6)	
Era of LT			0.260
2014–2016	933 (35.2)	3 (16.7)	
2017–2018	799 (30.1)	7 (38.9)	
2019–2020	920 (34.7)	8 (44.4)	
3.2 (2.8–3.7)	3.0 (2.6–3.6)	0.254	
Pre-transplant Total bilirubin (mg/dL, median)	2.2 (1.0–8.0)	1.9 (0.9–3.2)	0.491
Pre-transplant INR (median)	1.39 (1.15–1.95)	1.45 (1.20–1.97)	0.706
Pre-transplant Creatinine (mg/dL, median)	0.81 (0.64–1.07)	0.82 (0.62–1.00)	0.55
Ascites (n, %)			0.869
None	1020 (38.5)	8 (44.4)	
Mild (diuretic responsive)	844 (31.8)	5 (27.8)	
Moderate to severe (diuretic refractory)	788 (29.7)	5 (27.8)	
Hepatic encephalopathy (n, %)			0.838
None	2038 (76.8)	15 (83.3)	
Grade I–II	456 (17.2)	3 (16.7)	
Grade III–IV	158 (6.0)	0 (0.0)	
Child-Turcotte-Pugh (CTP) class (n, %)			0.455
A	792 (29.9)	5 (27.8)	
B	830 (31.3)	8 (44.4)	
C	1030 (38.8)	5 (27.8)	
CTP score (median)	8 (6–11)	8.5 (6–10)	0.637
MELD score (median)	14 (9–24)	12 (9–16.5)	0.335
Re-LT (n, %)			1
2nd re-LT	41 (1.5)	0 (0.0)	
3rd re-LT	5 (0.2)	0 (0.0)	
LT type (n, %)			0.011
Living donor	1991 (75.1)	18 (100.0)	
Deceased donor	661 (24.9)	0 (0.0)	
Donor characteristics			
Age (years, median)	36.4 (26.3–48.7)	32.6 (23.9–38.9)	0.027
Sex (n, % male)	1604 (60.5)	9 (50.0)	0.506
Relationship (n, %)			0.002
Parent	84 (3.2)	2 (11.1)	
Offspring	1266 (47.7)	15 (83.3)	
Sibling	199 (7.5)	0 (0.0)	
Spouse	277 (10.4)	0 (0.0)	
Relatives in blood	143 (5.4)	1 (5.6)	
Other relationships	683 (25.8)	0 (0.0)	
Graft weight (g, median)	710 (612–816)	764 (606–876)	0.383
GRWR (%, median)	1.09 (0.93–1.30)	1.18 (1.02–1.53)	0.093
Graft type (n, %)			0.045
Right lobe	1730 (65.2)	15 (83.3)	
Extended Right lobe	130 (4.9)	1 (5.6)	
Extended Left lobe	30 (1.1)	1 (5.6)	
Left lobe	58 (2.2)	0 (0.0)	
Left lateral section	59 (2.2)	1 (5.6)	
Whole liver	613 (23.1)	0 (0.0)	
Others	32 (1.2)	0 (0.0)	
Post-transplant outcome			
Warm ischemic time (min, median)	35.5 (27.0–49.0)	50.0 (36.5–61.0)	0.025
Cold ischemic time (hours, median)	1.9 (1.3–3.0)	1.9 (1.2–2.5)	0.615
GVHD (n, %)	7 (0.3)	4 (22.2)	< 0.001
Graft failure (n, %)	106 (4.0)	1 (5.6)	0.522
Biopsy proven Rejection (n, %)			0.353
Acute T-cell mediated rejection	267 (10.1)	0 (0.0)	
Acute antibody mediated rejection	13 (0.5)	0 (0.0)	
Chronic rejection	11 (0.4)	0 (0.0)	
Follow up period (years, median)	2.00 (0.49–4.00)	0.95 (0.25, 2.25)	0.060
Death (n, %)	307 (11.6)	7 (38.9)	0.003
Cause of death (n, %)			0.024
Cardiovascular disease	22 (7.2)	0 (0.0)	
Infection	126 (41.0)	2 (28.6)	
Liver failure	70 (22.8)	2 (28.6)	
Recurred HCC	45 (14.7)	1 (14.3)	
Malignancy except HCC	3 (1.0)	0 (0.0)	
GVHD	3(1.0)	2 (28.6)	
Others	38 (12.4)	0 (0.0)	

*PBC* primary biliary cirrhosis, *PSC* primary sclerosing cholangitis, *SSC* secondary sclerosing cholangitis, *HCC* hepatocellular carcinoma, *CCC* cholangiocellular carcinoma, *LT* liver transplantation, *MELD* Model for end-stage liver disease, *GRWR* graft-to-recipient weight ratio, *GVHD* graft-versus-host disease.

### HLA type comparison between donor and recipient with GVHD after LT

We analyzed HLA types at each locus (HLA-A, -B, -DR) of all 11 donors and recipients who were reported to develop GVHD after LT (Table 2). If they share the same HLA, it is highlighted with a gray box. Among 11 patients with GVHD after LT, 6 patients underwent living donor LT and all of them received liver grafts from their offspring. Four patients with living donor LT showed D → R one way HLA MM, and three had D → R one way HLA MM at 3 loci. All of the 5 patients who underwent deceased donor LT did not have D → R one way HLA MM.

**Table 2:** HLA types of GVHD patients and donor.

Type of LT	Recipient HLA						Donor HLA							
	A	A	B	B	DR	DR	A	A	B	B	DR	DR	R → D	D → R
													MM	MM
LD	31	33	44	54	12	13	11	33	44	54	4	13	2	2
	31	33	44	46	8	13	33	33	44	44	13	13	**0**	3*
	24	33	44	62	7	15	11	24	62	75	12	15	3	3
	2	2	60	61	8	14	2	2	61	61	8	14	**0**	1*
	24	33	44	46	7	8	33	33	44	44	7	7	**0**	3*
	2	31	51	62	4	15	31	31	62	62	15	15	**0**	3*
DD	2	29	61	75	1	14	1	24	27	51	1	8	5	5
	2	24	7	51	1	15	24	31	7	51	1	12	2	2
	2	24	35	51	8	9	1	3	35	37	1	10	5	5
	24	33	58	62	4	13	24	31	7	51	1	15	5	5
	2	31	35	71	9	12	2	2	71	71	4	15	2	4

*LD* living donor, *DD* deceased donor, *LT* liver transplantation, *R**** → ****D MM* recipient against donor HLA mismatch, *D**** → ****R MM* donor against recipient HLA mismatch.

*The values marked in bold are those for donor against recipient one-way HLA mismatch and are indeed significant values.

### Risk factors associated with GVHD, graft failure, patient death

We analyzed risk factors for GVHD by using multivariate logistic regression analysis (Table 3). In the multivariate analysis, only D → R one way HLA MM was found to be a strong independent risk factor of GVHD. In the presence of D → R one way HLA MM at 3 loci, the OR was calculated as 163.3 (95% CI = 34.15–780.89, *p* < 0.001) while the OR of 1&2 MM were 91.85 (95% CI = 8.98–939.32, *p* < 0.001).

**Table 3:** Risk factors associated with GVHD after LT (by Logistic regression analysis).

Variables	Univariate		Multivariate	
	OR (95% CI)	*p* value	OR (95% CI)	*p* value
Sex (female)	0.51 (0.11–2.35)	0.385		
Recipient age	1.03 (0.97–1.09)	0.323		
Hypertension	0.95 (0.2–4.41)	0.948		
Diabetes	1.59 (0.46–5.45)	0.461		
D → R one-way MM	1 (Ref.)		1 (Ref.)	
No one-way MM				
3 loci	141.7 (30.99–647.97)	< 0.001	163.3 (34.15–780.89)	< 0.001
1 and 2 loci	62.98 (6.68–593.46)	< 0.001	91.85 (8.98–939.32)	< 0.001
Recipient BMI	1 (0.91–1.11)	0.939		
CTP score	0.95 (0.75–1.19)	0.641		
MELD score	0.97 (0.91–1.04)	0.393		
LT type (LDLT vs. DDLT)	0.393	0.124		
Re-LT	0 (0, inf)	0.989		
GRWR	2.19 (0.77–6.20)	0.14		
Cold ischemic time	1.07 (0.97–1.19)	0.072	1.09 (1.00–1.20)	0.052
Warm ischemic time	1.01 (1–1.01)	0.146		

*MM* mismatch, *CTP* child-turcotte-pugh, *MELD* model for end-stage liver disease, *LDLT* living donor liver transplantation, *DDLT* deceased donor liver transplantation, *GRWR* graft-to-recipient weight ratio.

Risk factors associated with death-censored graft failure, and patient death were analyzed using Cox regression analysis (Table 4). Recipient age (HR = 0 0.98, 95% CI = 0.97–0.99, *p* < 0.001) and re-transplantation (HR = 2.76, 95% CI = 1.16–6.92, *p* = 0.005) were found to be associated with death-censored graft failure. However, D → R one way HLA MM was not associated with death-censored graft failure. In the multivariate analysis, deceased donor LT (HR = 1.64, 95% CI = 1.11–2.41, *p* = 0.012), D → R one way HLA MM at 3 loci (HR = 12.75, 95% CI = 5.16–31.52, *p* < 0.001), donor age (HR = 1.01, 95% CI = 1.00–1.02, *p* = 0.047), recipient age (HR = 1.03, 95% CI = 1.01–1.04, *p* = 0.047), MELD score (HR = 1.04, 95% CI = 1.02–1.06, *p* < 0.001), and re-transplantation (HR = 2.01, 95% CI = 1.05–3.87, *p* = 0.036) were found to be independent risk factors of patient death. However, D → R one way HLA MM at 1 or 2 loci was not related to either death-censored graft failure or patient death.

**Table 4:** Risk factors associated with graft failure and patient death (by Cox regression model).

Variables	Graft failure				Patient death			
	Univariate		Multivariate		Univariate		Multivariate	
	HR (95% CI)	*p* value	HR (95% CI)	*p* value	HR (95% CI)	*p* value	HR (95% CI)	*p* value
DDLT	1.6 (1.07–2.36)	0.021	1.37 (0.91–2.07)	0.133	2.36 (1.89–2.96)	< 0.001	1.64 (1.11–2.41)	0.012
D → R one-way MM	1 (Ref.)				1 (Ref.)		1 (Ref.)	
no one-way MM								
3 loci	2.98 (0.42–21.40)	0.277			5.20 (2.15–12.60)	< 0.001	12.75 (5.16–31.52)	< 0.001
1 & 2 loci	0 (0, Inf)	0.995			2.61 (0.65–10.50)	0.176	3.74 (0.52–26.93)	0.191
Donor age	1 (0.99–1.01)	0.962			1.02 (1.01–1.03)	< 0.001	1.01 (1.00–1.02)	0.047
Recipient age	0.98 (0.97–0.99)	< 0.001	0.98 (0.97–0.99)	< 0.001	1.01 (1–1.02)	0.056	1.03 (1.01–1.04)	< 0.001
Female recipient	1.24 (0.83–1.85)	0.286			1.1 (0.87–1.4)	0.422		
Recipient BMI	0.96 (0.92–1.01)	0.125			1 (0.98–1.02)	0.965		
Hypertension	1.32 (0.84–2.08)	0.235			1.39 (1.07–1.81)	0.014	1.17 (0.86–1.59)	0.311
Diabetes	0.84 90.53–1.33)	0.456			1.25 (0.98–1.59)	0.071	1.25 (0.94–1.65)	0.123
CTP score	1.02 (0.95–1.09)	0.64			1.14 (1.1–1.19)	< 0.001	0.97 (0.90–1.04)	0.421
MELD score	1.01 (1–1.03)	0.142			1.04 (1.03–1.05)	< 0.001	1.04 (1.02–1.06)	< 0.001
Re-LT	3.67 (1.49–9.01)	0.005	2.76 (1.16–6.92)	0.031	3.6 (2.11–6.15)	< 0.001	2.01 (1.05–3.87)	0.036
GRWR	0.96 (0.52–1.75)	0.884			1.09 (0.77–1.54)	0.627		
Cold ischemic time	1.03 (0.97–1.09)	0.391			1.06 (1.03–1.08)	< 0.001	0.99 (0.96–1.03)	0.713
Warm ischemic time	1 (0.99–1.01)	0.725			1 (1–1.01)	0.003	1.01 (1.00–1.01)	0.054
Rejection episode	0.84 (0.45–1.57)	0.591			0.77 (0.53–1.12)	0.176		

*MM* mismatch, *CTP* child-turcotte-pugh, *MELD* model for end-stage liver disease, *DDLT* deceased donor liver transplantation, *LT* liver transplantation, *GRWR* graft-to-recipient weight ratio.

### Clinical courses and outcomes of cases with donor against recipient one-way HLA mismatch

Table 5 summarizes the clinical courses of 18 cases with D → R one way HLA MM after LT. The cases were classified according to the number of HLA mismatches at each locus. There were 11 cases of D → R one way HLA MM at 3 loci, 2 cases of D → R one way HLA MM at 2 loci, and 5 cases of D → R one way HLA MM at 1 locus. In cases with mismatch at 3 loci, all the donors’ HLA type showed homozygous pattern. Five out of 11 recipients with D → R one way HLA MM at 3 loci died within a year after LT and among them, GVHD was reported in 3 cases, all of which developed 1–2 months after transplantation. Among 5 cases of D → R one way HLA MM at 1 locus, 2 recipients died, and one of them was reported to have GVHD.

**Table 5:** Clinical courses of cases of donor against recipient one-way HLA mismatch.

No. of D → R one way MM	Case no	Sex/Age	LT type (relationship)	HLA type				Reason for LT	MELD/PELD	GRWR (%)	Recurrence of liver disease	Surgical complication	Death (duration after LT)	Cause of death
				A	B	DR	DQ							
**3**	1													
	Recipient	M/61	LDLT	24, 33	44, 62	7, 12		HCC-B (TACE/RFA)	11	0.75		Bile duct stenosis & leakage/stone	Yes (10.7 months)	Graft failure d/t biliary complication
	Donor	F/25	(Offspring)	33, –	44, –	7, –								
	2													
	Recipient	M/41	LDLT	31, 33	44, 46	8, 13		Alcoholic LC	18	1.71		Yes	Yes (1.9 months)	GVHD
	Donor	F/20	(Offspring)	33, –	44, –	13, –								
	3													
	Recipient	M/65	LDLT	2, 33	44, 62	4, 13		HCC-C (TACE)	9	1.17	HCV	No	No	
	Donor	M/40	(Offspring)	33, –	44, –	13, –								
	4													
	Recipient	M/54	LDLT	31, 33	44, 60	4, 7		HCC-B	13	1.02		No	No	
	Donor	F/26	(Offspring)	33, –	44, –	7, –								
	5													
	Recipient	M/66	LDLT	24, 33	44, 46	7, 8		HCC (beyond Milan)	10	1.13		Bile leakage	Yes (1.0 months)	GVHD
	Donor	F/38	(Offspring)	33, –	44, –	7, –								
	6													
	Recipient	M/53	LDLT	11, 33	44, 62	4, 7		HCC-B (TACE)	10	1.38	HCC-B	No	Yes (10.6 months)	Infection
	Donor	M/28	(Offspring)	11, –	62, –	4, –								
	7													
	Recipient	M/66	LDLT	26, 33	44, 54	13, 14		Alcoholic LC, HCC (beyond Milan)	15	1.18		No	Unknown (2.9 months)	
	Donor	M/39	(Offspring)	33, –	44, –	13, –								
	8													
	Recipient	F/57	LDLT	24, 33	51, 58	13, 14		Alcoholic LC	6	1.34		No	No	
	Donor	F/33	(Offspring)	33, –	58, –	13, –								
	9													
	Recipient	M/64	LDLT	2, 31	51, 62	4, 15		Alcoholic LC, HCC (TACE)	11	1.79		No	Yes (1.0 months)	GVHD
	Donor	M/34	(Offspring)	31, –	62, –	15, –								
	10													
	Recipient	F/10	LDLT	26, 33	37, 44	7, 10		Biliary atresia	1	1.32		No	No	
	Donor	F/43	(Parent)	33, –	44, –	7, –								
	11													
	Recipient	F/33	LDLT	24, 33	44, 59	4, 13		Alcoholic LC	14	1.33		No	No	
	Donor	F/32	(Relatives)	33, –	44, –	13, –								
**2**	12													
	Recipient	F/1	LDLT	2, 24	7, 51	1, 15		Budd-Chiari syndrome	11	2.78	Hepatic vein stenosis/thrombosis (3 yr after LT)	No	No	
	Donor	F/34	(Parent)	2, 24	7, –	1, –								
	13													
	Recipient	F/67	LDLT	2, –	50, 61	7, 15		Drug toxicity	29	0.94		No	No	
	Donor	M/39	(Offspring)	2, –	61, –	15, –								
**1**	14													
	Recipient	M/48	LDLT	2, –	60, 61	8, 14	5, 6	HCC-B (Liver resection/ TACE/CCRT)	15	1.01		No	Yes (3.1 months)	GVHD
	Donor	M/17	(Offspring)	2, –	61, –	8, 14	5, 6							
	15													
	Recipient	F/49	LDLT	3, 33	44, –	13, –		Alcoholic LC	9	1.68		No	No	
	Donor	M/24	(Offspring)	33, –	44, –	13, –								
	16													
	Recipient	M/47	LDLT	2, 24	52, 62	14, 15		HCC-B (TACE/beyond Milan)	32	–	HCC-B	No	Yes (47.0 months)	Recurred HCC
	Donor	F/17	(Offspring)	2, 24	62, –	14, 15								
	17													
	Recipient	F/51	LDLT	3, 33	44, 58	4, 13		Unknown	17	1.07		No	No	
	Donor	M/20	(Offspring)	3, 33	44, 58	13, –								
	18													
	Recipient	M/57	LDLT	24, 26	7, 54	1, 4		HCC-B (Liver resection/TACE)	9	0.82		Hepatic artery stenosis Post op bleeding	No	
	Donor	M/33	(Offspring)	24, –	7, 54	1, 4								

*LDLT* living donor liver transplantation, *DDLT* deceased donor liver transplantation, *HCC* hepatocellular carcinoma, *LC* liver cirrhosis, *GRWR* graft-to-recipient weight ratio, *GVHD* graft-versus-host disease, *RFA* radiofrequency ablation, *TACE* transcatheter arterial chemoembolization.

**Figure 2:**
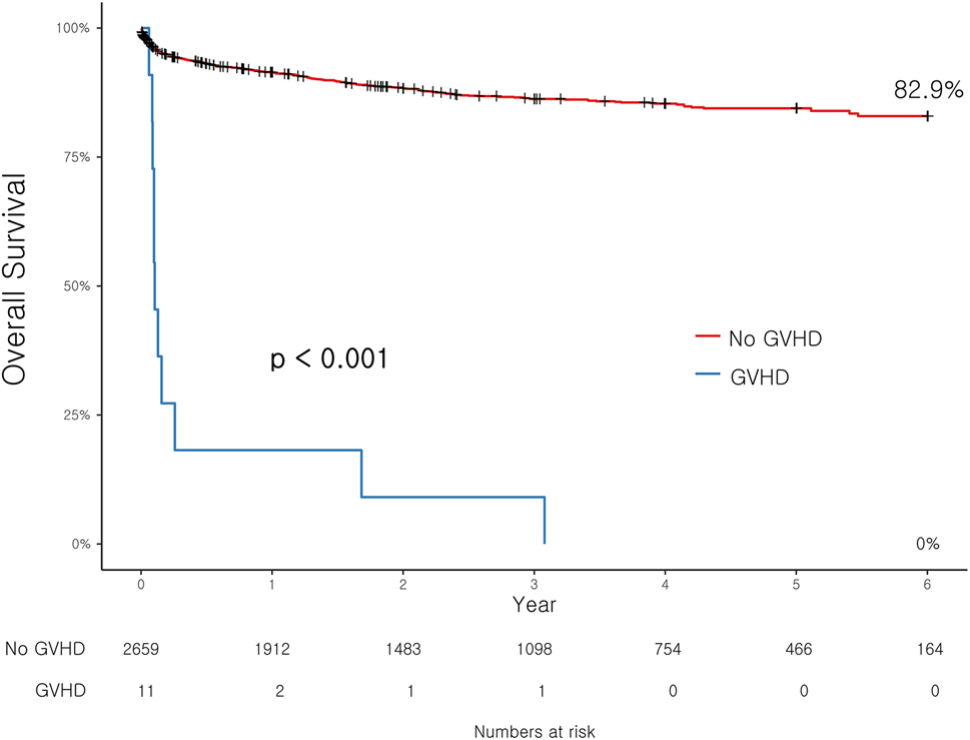
Pateint surival based on the occurrence of GVHD after liver transplantation.

**Figure 3:**
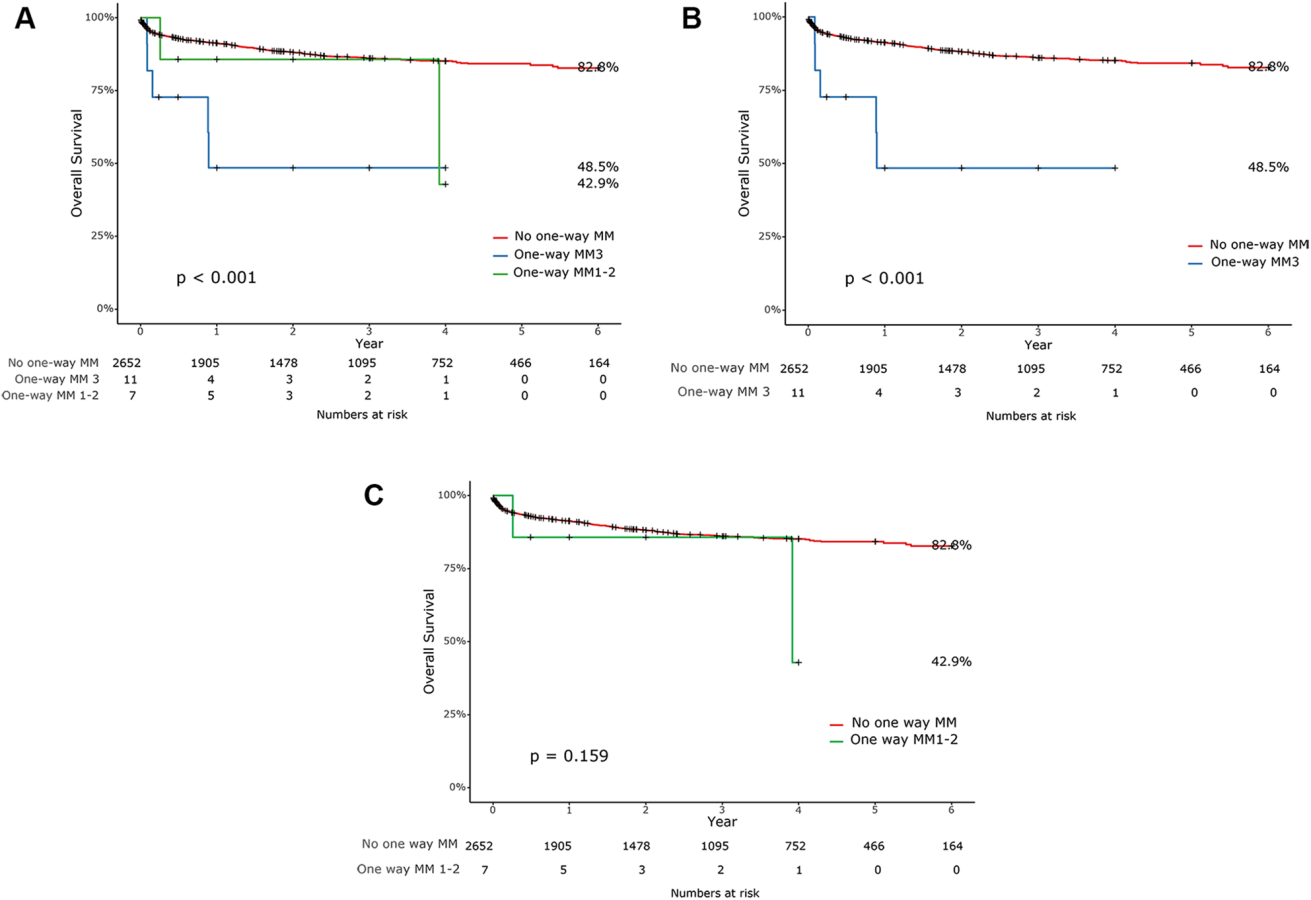
Patient survival according to the number of donor against recipient one-way HLA mismatch (**A**). Patient survival between control group and donor against recipient one-way HLA mismatch at 3 loci (**B**). Patient survival between control group and donor against recipient one-way mismatch at 1 or 2 loci (**C**).

**Figure 4:**
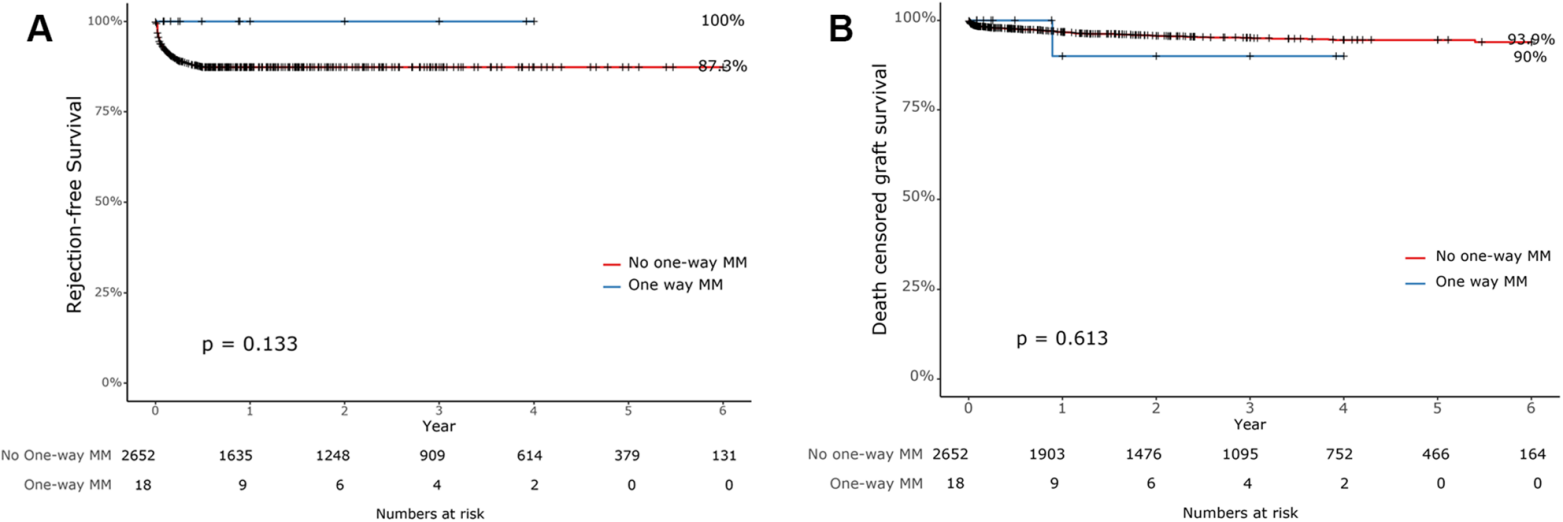
Comparison of rejection-free survival (**A**) and death-censored graft survival (**B**) between donor against recipient one-way HLA mismatch group and the control group.

Figure 2 displays the survival curve according to the presence or absence of GVHD, and it is evident that patients who developed GVHD after liver transplantation showed significantly lower patient survival compared to those who did not. The patient survival, rejection free survival, and death-censored graft survival were also evaluated according to the number of HLA mismatches. D → R one-way HLA MM group again divided into two groups as patients with one-way MM at 3 loci and those with one-way MM at 1 or 2 loci. Patients with D → R one-way HLA MM at 3 loci showed significantly lower patient survival comparing to the control group (*p* < 0.001), while patients with D → R one-way HLA MM at 1 or 2 loci showed no difference against the control group (*p* = 0.159) (Fig. 3). Patient survival between D → R one-way HLA MM at 3 loci group and D → R one-way HLA MM at 1 or 2 loci group also showed no difference (*p* = 0.297). There were no significant differences in rejection-free survival (*p* = 0.133) and death-censored graft survival (*p* = 0.613) according to the number of D → R one-way HLA MM (Fig. 4).

## Discussion

There have been several studies on the impact of HLA compatibility on the outcomes after LT. According to some previous studies, it was obvious that the higher the HLA compatibility, the lower the probability of acute rejection after LT, but the effect on overall graft survival has been controversial^2,9^. Donaldson et al.^3^, demonstrated that HLA class I matching may affect the graft survival by increasing the possibility of development of acute rejection and the vanishing bile duct syndrome. However, by analyzing the OPTN database, Navarro et al.^10^ showed that there has been no clinically significant relationship between the degree of HLA matching and 5 years graft survival of LT. Recently, Bricogne et al.^11^ found that locus-specific HLA-A mismatching was associated with reducing graft failure and patient survival after LT by increasing hepatic artery thrombosis and sepsis.

There also has been an effort on finding relationship between HLA compatibility and GVHD after LT. Some previous studies highlighted the importance of D → R one-way HLA MM as a strong risk factor for GVHD^12,13^. However, since the prevalence of GVHD is extremely low, studies on the topic have been very difficult to find in the past decades. The previous study published by Kim et al.^14^, a retrospective single-center study, indicated that the D → R one-way HLA MM at 3 loci significantly increased the incidence of GVHD after LT. However, our study was designed as a large-scale multi-institutional study that used a prospectively updated data from a KOTRY registry. By comparing the D → R one-way HLA MM patient group with no D → R one-way HLA MM patient group, this study revealed a significant difference in the incidence of GVHD between these two groups. Moreover, for the D → R one-way HLA MM patient group, further division was made based on the number of MM loci differences to analyze the risk of GVHD, graft failure, and patient survival. Thereby showing that this phenomenon is not restricted to a single center but has similar outcomes in other centers.

Since this study is based on KOTRY data, it is difficult to review the clinical course of each patient. Therefore, there is a possibility of underdiagnosing GVHD in the study population. However, even considering the omission of some GVHD patients due to underdiagnosed GVHD, it was found that the D → R one-way HLA MM group showed a much higher incidence rate. About 0.4% (11 out of 2670 patients) of all the study cohort in the study was reported to have GVHD after LT. A total of 22.2% of patients were reported to have GVHD in D → R one-way HLA MM group, while in the control group, only 0.3% of the patients were reported to have GVHD. To more accurately analyze the relationship between GVHD and D → R one-way HLA MM, we analyzed HLA type at each locus (HLA-A, -B, -DR) of all patients with GVHD after LT. Interestingly, 4 out of 6 cases of living donor LT with GVHD showed D → R one-way HLA MM but none of the deceased donor LT cases with GVHD showed D → R one-way HLA MM. Especially, D → R one-way HLA MM at 3 loci occurred when the HLA-homozygous donor shared haplotype with HLA-heterogenous recipient, and this combination of HLA type usually happened when transplantation was made between parent and child. In fact, in the present study, all recipients with GVHD after living donor LT received liver graft from their offspring, and D → R one-way HLA MM was seen in a large proportion, which was 66.7% of recipients with GVHD after living donor LT.

In previous studies, close matching of HLA types between donors and recipients, age of recipients, and glucose intolerance found out to be risk factors for GVHD after LT^4,6,8^. However, in our study, D → R one-way HLA MM was the only significant independent risk factor for GVHD after LT. In multivariate analysis, D → R one-way HLA MM at 3 loci and D → R one-way HLA MM at 1 or 2 loci seemed highly associated with GVHD with odd ratio of 163.3 and 91.85, respectively. On the other hand, D → R one-way HLA MM was not related to graft failure after LT, and D → R one-way HLA MM at 1 or 2 loci was found not to be a risk factor for patient death. Only D → R one-way HLA MM at 3 loci was identified as the strongest risk factor for patient death. These findings represent that D → R one-way HLA MM at 3 loci is one of the most potent risk factors for GVHD which could lead to a devastating outcome such as patient death. In addition, according to the Kaplan–meier survival curve analysis, not D → R one-way HLA MM at 1 or 2 loci but only D → R one-way HLA MM at 3 loci showed significant lower patient survival compared to those who do not have D → R one-way HLA MM. There were no significant differences in death-censored graft survival between D → R one-way HLA MM group and the control group. In terms of rejection-free survival, there was no statistically significant difference between the two groups. However, it was observed that there was no incidence of rejection in the patient group with D → R one-way HLA MM. From the perspective of the recipient in the D → R one-way HLA MM situation, there's no mismatch with the donor's HLA, implying a very low possibility of the existence of donor-specific antibodies (DSA). This can be seen as a result that supports previous research findings suggesting that DSAs can influence rejection after liver transplantation^15–18^.

The limitation of this study is that since it is a retrospective multicenter study using the KOTRY data, information on other types of HLA alleles such as HLA-C, -DQ, and -DP were lacking, making it difficult to analyze on those HLA alleles. Likewise, it was unavailable to use the latest and more precise PCR based HLA typing data. Prior research in hematopoietic stem cell transplantation indicated that an antigen mismatch at the HLA-C * 14:02 locus might elevate the risk of GVHD, implying that a similar mismatch of antigens could also adversely affect liver transplantation outcomes^19^. However, Hirata et al.^20^ reported that the influence of HLA-C, HLA-DQ, and HLA-DP on GVHD was not as pronounced as that of HLA-A, HLA-B, and HLA-DR. We anticipate that with the accumulation of additional data on high-resolution sequence-based HLA typing and epitope matching in the future, we can proceed with further research and potentially undertake a more accurate mismatch analysis which may influence the outcome of liver transplantation, especially for GVHD. It was also hard to review the specific clinical course of each case from KOTRY data. Therefore, due to the non-specific symptoms and difficulties in making a definitive diagnosis of GVHD, there might be cases which were omitted to be diagnosed as GVHD. However, prospectively data collecting multicenter data clearly showed that HLA type, specifically, D → R one-way HLA MM at 3 loci is an extremely lethal prognostic factor for the occurrence of both GVHD and patient death which have its strength in generalizability of the finding.

Although there have been some studies reporting the risk of D → R one-way HLA MM at 3 loci on GVHD, we can assume that the importance is not quite emphasized to the general field of LT, since there are still cases with living donor LT with D → R one-way HLA MM. This may be due to the minimal reporting data on the topic since the odds are very low even in living donor LT setting. Before the LT community find the solution for this devastating condition, the only solution is not to perform transplantation in this high-risk combination of donor and recipient.

In conclusion, among patients considering living donor LT, if donors’ HLA type shows homozygous pattern and shares haplotype of HLA with their recipients, the probability of D → R one-way HLA MM at 3 loci would be very high, which is associated with GVHD. This could lead to fatal outcome, leading to the death of the patient. Therefore, in such a case, it is necessary to select alternative donor or to withhold transplantation. However, although the possibility of developing D → R one-way HLA MM is extremely rare in the case of deceased donor LT, there is still possibility that GVHD can occur so that further research should be needed on GVHD which occurs during deceased donor LT.

## Patients and methods

### Study cohort

This study is a retrospective cohort study of patients enrolled in KOTRY database. KOTRY is a prospective, multicenter cohort registry, which is composed of 5 solid organ transplantation cohorts, including kidney, liver, pancreas, heart, and lung^21^. Patients who underwent LT from 15 institutions in South Korea are enrolled in KOTRY. After receiving written informed consent prior to transplantation, patients were registered in the KOTRY database, and routinely followed-up. This study was reviewed and approved by the Institutional Ethics Committees of each participating center, according to the Regulations on Human Organ Transplant and national legal requirements. The procedures were in accordance with the Helsinki declaration.

We reviewed 5105 patients who underwent LT between April 2014 and December 2020 in KOTRY database. In South Korea, the HLA typing test of LT patients was not yet implemented in earnest until 2016, and since it was a retrospective study using the KOTRY data, HLA typing test results were often omitted. Therefore, among them, 2670 patients with serologic HLA typing of both recipient and donor were included in this study while who do not have the data were excluded. The cohort was divided into two groups according to the presence of D → R one-way HLA MM. The diagnosis of GVHD in the study was based on the reports of the participating centers.

### Diagnosis criteria of GVHD

When clinical symptoms such as skin rash, cytopenia, or diarrhea were observed after a liver transplant, raising suspicions of GVHD, a pathological biopsy (confirmed through methods like skin biopsy or mucosal biopsy via colonoscopy) was conducted. Whenever possible, positive chimerism of the peripheral blood was also performed to diagnose GVHD. The diagnostic approach was carried out in accordance with the protocols of each hospital.

### Definition of donor against recipient one-way HLA mismatch (D → R one-way HLA MM)

Serologic HLA typing with HLA-A, -B, -DR locus-specific analysis of both recipient and donor was performed before transplantation. The number of serologic HLA mismatches on each locus between donors and recipients were identified. When all the donor’s HLA types were included in recipient’s HLAs while some of the recipient’s HLAs were different from the donors’ HLAs, the condition was defined as donor against recipient one-way HLA mismatch (D → R one-way HLA MM).

### Statistical analysis

Numerical variables of the two groups were presented as median with interquartile range (IQR) which were analyzed by Mann–Whitney test. Categorical variables of the two groups were presented as percentage and were analyzed by Pearson’s chi-squared test or Fischer’s exact test. Logistic regression analysis was used for analyzing risk factors of GVHD. Univariate and multivariate Cox proportional hazard regression model was used in analyzing risk factors associated with graft failure, and patient death after LT. Kaplan–Meier survival curve analysis was performed to estimate the overall patient survival, rejection-free survival, and death-censored graft survival between the two groups. Statistical analyses were conducted with SPSS 26.0 (SPSS Inc., Chicago, IL).

## Data Availability

The datasets generated and analyzed during the current study are available from the corresponding author on reasonable request.

## Abbreviations

LT: Liver transplantation
GVHD: Graft-versus-host disease
HLA: Human leukocyte antigen
LDLT: Living donor liver transplantation
GRWR: Graft-recipient weight ratio
IQR: Interquartile range
MELD: Model for end-stage liver disease
DDLT: Deceased donor liver transplantation
